# Bioactive Self-Polymerizing Resin with Surface Pre-Reacted Glass Ionomer Fillers for Suppressed Enamel Demineralization

**DOI:** 10.3390/ma17205101

**Published:** 2024-10-18

**Authors:** Naoyuki Kaga, Masayuki Kaga, Sho Morita, Futami Nagano-Takebe, Takashi Nezu, Kazuhiko Endo, Takashi Matsuura

**Affiliations:** 1 Section of Fixed Prosthodontics, Department of Oral Rehabilitation, Fukuoka Dental College, Fukuoka 814-0193, Japan; moritas@fdcnet.ac.jp (S.M.); matsuurt@fdcnet.ac.jp (T.M.); 2 Oral Medicine Research Center, Fukuoka Dental College, Fukuoka 814-0193, Japan; 3 Division of Biomaterials and Bioengineering, Department of Oral Rehabilitation, School of Dentistry, Health Sciences University of Hokkaido, Ishikari-Tobetsu 061-0293, Japan; sp8c5fx9@salsa.ocn.ne.jp (M.K.); nagano23@hoku-iryo-u.ac.jp (F.N.-T.); tnezu@hoku-iryo-u.ac.jp (T.N.); endo@hoku-iryo-u.ac.jp (K.E.)

**Keywords:** auto-polymerizing resin, S-PRG filler, bioactivity, ion release, acid buffering capacity, enamel demineralization, provisional restoration

## Abstract

The treatment of damaged enamel surfaces involves modification of the enamel surface with artificial materials or the development of a pseudo-enamel, with research focusing on bioactive and biomimetic materials. In this study, a bioactive auto-polymerizing resin (APR) was developed by adding surface-pre-reacted glass ionomer (S-PRG) fillers of different quantities to APR. Its bioactive effects were evaluated via pH neutralization, ion release, and inhibition of enamel demineralization studies. The pH and fluoride ion release were measured using ion-specific electrodes, revealing that the APR disk with the S-PRG filler immediately neutralized the lactic acid solution (pH 4.0) through ion release. Inductively coupled plasma atomic emission spectrometry revealed that the Sr ion release peaked on the first day, with the other ions following the order F > B > Si > Al > Na, exhibiting a weekly decrease in the same order. Scanning electron microscopy was used to examine the enamel block morphology of the disks after 7 d of incubation, revealing enamel demineralization in disks without the S-PRG filler, whereas no demineralization occurred in disks with the S-PRG filler. APR containing the S-PRG filler demonstrated acid buffering suppressed enamel demineralization and bioactive properties.

## 1. Introduction

The number of elderly people who use removable partial dentures is increasing annually, requiring home nursing care and bedridden dental treatment. Furthermore, the demand for home-visit dental care throughout one’s lifespan is growing, as dental care may not be easily accessible to the elderly and patients with impaired cognitive or motor function. These people are unable to clean themselves properly, with denture cleaning being insufficient. Elderly people who wear dentures tend to have poor oral hygiene and suffer from caries, periodontitis, and denture stomatitis [1,2,3,4]. Dental plaque tends to accumulate, particularly on the teeth supporting clasps and in regions where the denture base resin covers both teeth and soft tissues. Additionally, the clasp and denture base impede physiological clearance, leading to food accumulation, providing space for bacterial adhesion, and increasing the risk of caries on denture abutment teeth [5,6]. The treatment of caries in elderly patients who wear dentures and do not maintain proper oral hygiene is accompanied by difficulties in moisture control and poor adhesion to the tooth substrate. Conventional treatments involve mechanical removal of decayed lesions and fillings; however, these methods are inadequate in cases involving extensive enamel erosion or root caries. Biological and technological advances in dental materials have led to non-invasive oral cavity treatments that involve controlling caries initiation and remineralizing the early stages of caries. These materials should exhibit sufficient quality to repair diseased or damaged tooth structures in hard tissues [7,8], with the main goal being finding alternative materials or methodologies to restore or reconstruct the enamel.

Efforts to treat damaged enamel surfaces include modifying the enamel surface with artificial materials or developing a pseudo-enamel, with current research shifting from biocompatible to bioactive and biomimetic materials. The tooth surface is chemically modified with antibacterial agents and sustained-release ions, which cause lethal damage to oral bacteria that adhere to the teeth and inhibit biofilm formation. This prevents bacteria from adhering to the tooth surface and inhibits tooth demineralization caused by bacterial acid production, thereby suppressing dental caries and oral bacterial diseases. Efficient denture materials mixed with fillers should reduce the development of adherent biofilms, denture caries, and stomatitis through proper denture hygiene. In a previous study, acrylic denture bases with surface pre-reacted glass ionomer (S-PRG) fillers or fluorinated glass fillers significantly reduced *C. albicans* adhesion without altering the mechanical properties of the resins [9]. S-PRG fillers are a novel kind of particles that exert bioactive effects with a stable glass ionomer phase that releases aluminum (Al), boron (B), sodium (Na), silicate (Si), and strontium (Sr) ions, being promising as functional biomaterials [10]. S-PRG fillers are currently used as preventive or restorative materials in commercially available dental products; however, they have not yet been applied to denture prosthodontic materials or provisional restorations [11,12]. In vitro studies using enamel specimens and dental materials containing S-PRG fillers have proven their effectiveness in preventing enamel or dentin demineralization [13,14,15,16]. Furthermore, the eluates of S-PRG fillers inhibit the growth activity of the caries-oriented acid-producing bacteria *S. mutans* [17,18,19]. Therefore, it is important to develop denture materials that automatically release ions when worn in the patient’s mouth to prevent the attachment of cariogenic bacteria or *Candida* and their acid neutralization ability.

In this study, an experimental model was devised to determine the effectiveness of S-PRG fillers in terms of pH neutralization, ion release, and inhibition of enamel demineralization in acidic environments. The objective was to explore the effects of incorporating auto-polymerizing resin (APR) with an S-PRG filler as a bioactive prosthetic material in the development of a novel denture material exhibiting biological functions to prevent enamel demineralization. An experimental model was developed to determine the effects of S-PRG fillers on pH neutralization, ion release, and inhibition of enamel demineralization in acidic environments. The null hypothesis was that this new APR formed with S-PRG filler would not exhibit sufficient bioactivity in terms of clinical performance.

## 2. Materials and Methods

### 2.1. Study Design and Methodology

**Figure 1:**
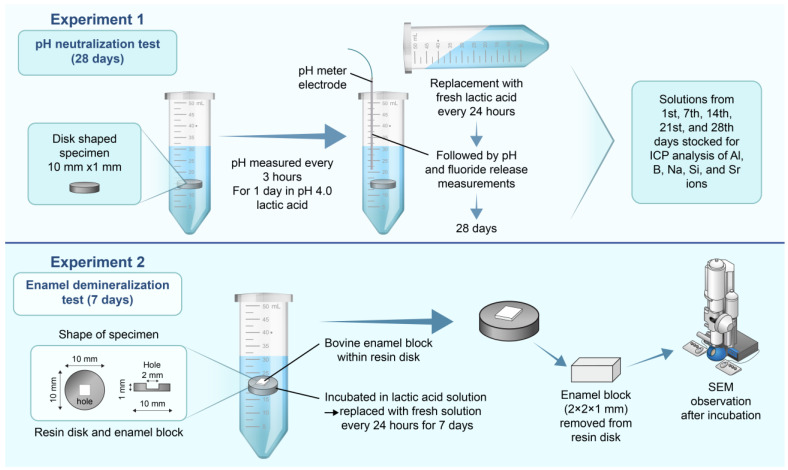
Schematic diagram of the study design and methodology. Experiment 1: pH neutralization test. Experiment 2: Enamel demineralization test apparatus in which the enamel block was attached to a disk specimen containing the S-PRG filler.

Figure 1 shows a schematic of the experimental procedure and methodology employed in this study.

### 2.2. SEM Microstructural Analysis of the Auto-Polymerizing Resin Powder and S-PRG Filler

APR powder (polymers: Provinice, 3S, Shofu Inc., Kyoto, Japan) and S-PRG filler (Shofu Inc., Kyoto, Japan), with a mean particle size of approximately 3 μm, were used in this study. The resin powder and S-PRG filler were sputter-coated with Au-Pt and were observed under a scanning electron microscope (SEM; JEOL JSM-6330F, JEOL, Tokyo, Japan) at a 5 kV acceleration voltage.

### 2.3. Preparation of Disk-Shaped Specimens

Experiment 1 (Figure 1) shows the methodology followed for the acid neutralization and ion release analyses. APR was mixed with the S-PRG filler at 0, 10, 20, 30, and 40 wt% using a vortex mixer (NS-80, AS ONE Inc., Osaka, Japan) for 10 min following the method described in a previous study, with modifications [13]. The prepared samples served as the experimental groups, whereas those without the S-PRG filler (0 wt%) served as the control group. Disk-shaped specimens were prepared for acid buffering and enamel demineralization tests (10 mm diameter, 1 mm thickness, n = 6). The APR powder containing the S-PRG filler and liquid (monomer, Shofu Inc., Kyoto, Japan) was kneaded following the manufacturer’s directions (powder to liquid ratio of 1.0 g/0.5 mL) at room temperature (24 ± 1 °C) and was filled into silicone molds (10 mm in diameter × 1 mm in heigh) after having a doughy consistency. The specimens were overlaid on a polyethylene sheet, pressed onto a metal sheet, and maintained at room temperature for 30 min. The specimens were then separated from the silicon molds and kept in a 37 °C incubator for 24 h prior to the experiments.

### 2.4. Acid Neutralization Capacity

A DL-lactic acid solution (Wako Pure Chemical Industries, Ltd., Osaka, Japan) was prepared via dilution with distilled water to a concentration of 0.2 mL, resulting in a pH of 4.0. The lactic acid solution (5 mL) was placed into 50 mL centrifuge tubes (AGC TECHNO GLASS Co., Ltd., Shizuoka, Japan) followed by addition of the disk-shaped specimens with the S-PRG filler and storage at 37 °C. The pH of each solution was measured every 3 h for up to 24 h. Subsequently, the disk-shaped specimens were placed in centrifuge tubes filled with fresh lactic acid solutions of pH 4.0 and left at 37 °C for 24 h. This procedure was performed every 24 h for 28 d to confirm the long-term durability of the samples. An Orion 8102BNUWP pH electrode (Thermo Fisher Scientific, Waltham, MA, USA) was linked to a pH/ion meter (Orion 2115010 Dual Star pH/ion meter, Thermo Fisher Scientific, Waltham, MA, USA) and placed in the middle of a centrifuge tube containing the lactate solution and a disk-shaped specimen for pH measurements (Figure 1, Experiment 1).

### 2.5. Ion Release Analysis

#### 2.5.1. Fluoride Release

After acid neutralization every 24 h for 28 d, the concentration of the fluoride ions emitted from the disk-shaped specimen was determined using a fluoride ion-selective electrode (Orion 9609 BNWP, Thermo Fisher Scientific, Waltham, MA, USA) in conjunction with an ion-analyzer (Orion 2115010 Dual Star pH/ion meter, Thermo Fisher Scientific, Waltham, MA, USA). To accurately determine the fluoride concentration, 1 mL of the lactic acid solution was withdrawn and 10 vol% TISAB III buffer was added to decomplex the fluoride complex. The mixed solution was agitated gently for 15 min and the fluoride ions were measured.

#### 2.5.2. Al, B, Na, Si, and Sr Ions Analysis

The disk-shaped specimens were immersed in the lactic acid solution for 1, 7, 14, 21, and 28 d, followed by measurement of the ions released into the solution. Elemental analyses of the released Al, B, Na, Si, and Sr ions were conducted using inductively coupled plasma-optical emission spectroscopy (ICP-OES) with an Optima 5300 DV system (Perkin-Elmer, Waltham, MA, USA). Calibration curves corresponding to each element were prepared and analyzed. The target calibration standards were set at 0.1, 1.0 and 10.0 ppm for each ion. While deionized water and lactic acid solution (pH 4.0) were used as the controls.

### 2.6. Preparation of Resin Disk and Enamel Block Specimens

Experiment 2 in Figure 1 shows the experimental methodology and enamel demineralization test apparatus in which the enamel block was attached to a disk specimen containing the S-PRG filler.

#### 2.6.1. Enamel Block Preparation

A bovine incisor (Yokohama Meat Corporation, Yokohama, Japan) free of lesions and cracks was used. The enamel of bovine incisors is a nearly completely mineralized tissue consisting of hydroxyapatite, which has a chemical composition of Ca_10_(PO_4_)_6_(OH)_2_. First, the crowns were separated from their roots and flat enamel surfaces on the buccal side of bovine incisors were cut to a thickness of 1 mm using a low-speed diamond saw (Isomet, Buehler, Lake Bluff, IL, USA) and running water. The previously-exposed enamel surfaces were carefully polished using a waterproof 2000-grit silicon carbide abrasive paper (Sankyo Rikagaku Co., Ltd., Saitama, Japan) and submerged in running water to enhance the surface smoothness. The enamel blocks were prepared by applying an acid-resistant nail varnish to the dentin side of the slabs, followed by cutting them into uniform 2 mm × 2 mm pieces using a diamond saw.

#### 2.6.2. Enamel Demineralization Test

For the enamel demineralization test, a silicone mold was created with a 2 mm × 2 mm × 0.8 mm space in the center of a disk specimen (10 mm diameter × 1.0 mm thickness) to accommodate an enamel block (approximately 2 mm × 2 mm × 1 mm). The mold was filled with kneaded resin to prepare an enamel demineralization test specimen, in which an enamel block was placed in the middle of the disk-shaped specimen. The pH of the DL-lactic acid solution was adjusted to 4.0 and the procedure was carried out according to the method described above (Figure 1, Experiment 2). The specimens, consisting of enamel blocks embedded in APR disks containing S-PRG fillers, were immersed in the lactic acid solution (pH 4.0) and stored at 37 °C. After 24 h, the specimens were transferred to a centrifuge tube containing a fresh pH 4.0 lactic acid solution, and stored at 37 °C for another 24 h; this process was repeated for 7 d. After 7 d of incubation, the enamel blocks were removed from the disk for examination via SEM. The blocks were first washed with distilled water and then dehydrated in a graded ethanol solution (60–100%). Subsequently, the blocks were sputter-coated with a layer of Au-Pt and observed via SEM at an acceleration voltage of 5 kV.

### 2.7. Statistical Analysis

All statistical analyses were performed using GraphPad Prism version 8.1.2 (GraphPad Software Inc., La Jolla, CA, USA). Normality and homogeneity of variance of the data were confirmed in advance using the omnibus K2 test. The impact of the S-PRG filler content and incubation time on the pH levels and release profiles of the F, Al, B, Na, Si, and Sr ions was assessed using two-way ANOVA and Dunnett’s multiple comparison test. The control group was APR with 0 wt% S-PRG filler, and the significance level was set at 0.05, with a *p*-value of less than 0.05. Representative areas of the enamel block surface were randomly selected and observed using SEM.

## 3. Results

### 3.1. Microstructural Analysis of the S-PRG Filler and Auto-Polymerizing Resin Powder

**Figure 2:**
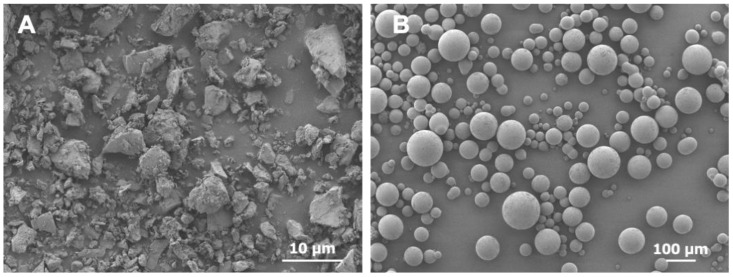
Representative SEM images of the (**A**) S-PRG filler (average particle size of 3 μm, ×2000) and (**B**) auto-polymerizing resin powder (polymer, ×100).

Figure 2 shows a representative SEM image of the S-PRG filler and the auto-polymerizing resin powder (polymer). The S-PRG filler exhibited a plate-like shape of approximately 3 μm and different sizes (Figure 2A). The resin polymer exhibited a spherical shape of approximately 50 μm (Figure 2B).

### 3.2. Variations in Acid Neutralization Capacity

**Figure 3:**
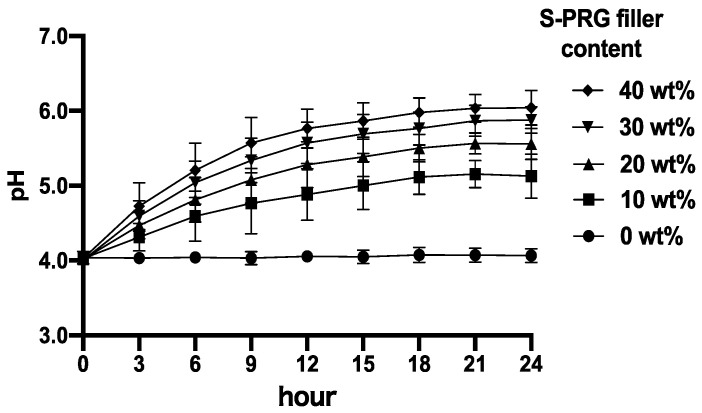
pH curves of the five S-PRG filler-containing auto-polymerizing resin disks immersed in lactic acid solution for 24 h.

Figure 3 shows the serial curves of the pH change within 24 h obtained by periodic pH measurements every 3 h. In the APR without the S-PRG filler (control), the pH remained at its initial value (pH 4.0) for 24 h. pH measurements after 3 h showed pH values of 4.4, 4.5, 4.7, and 4.8 for the APR with 10, 20, 30, and 40 wt% S-PRG fillers, respectively. After 9 h, the pH value steadily increased to >5.1 in all disks except for APR with 10% S-PRG filler. Thereafter, the pH gradually increased toward the neutral range in the solutions containing all APRs with the S-PRG filler, ranging from 5.1 to 6.1 after 24 h. The pH increased as the amount of the S-PRG filler increased.

**Figure 4:**
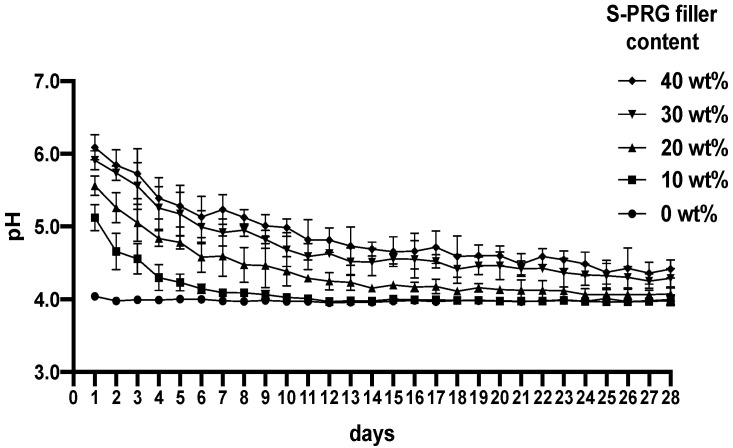
pH curves of the five S-PRG filler-containing auto-polymerizing resin disks immersed in lactic acid solution for 28 d.

Figure 4 shows the pH changes of the lactic acid solutions containing the APRs and S-PRG filler disks incubated for 24 h to 28 d. The pH of each solution containing the disk was the highest at 24 h and ranged from 5.1 to 6.1 for APR disks with 10–40 wt% S-PRG filler. The acid-neutralizing capacity gradually decreased over time up to 28 d, following the same trend as the amount of the S-PRG filler. APR with 10 and 20 wt% S-PRG fillers lost their neutralizing capacity at pH 4.1 on the 7th and 12th day, respectively. However, APR with 30 and 40 wt% S-PRG filler maintained its neutralizing capacity, exhibiting a pH value of 4.3 and 4.4, respectively, on day 28. Table 1 shows the statistical analysis of the pH values at days 1, 7, 14, 21, and 28.

### 3.3. Ion Analysis

#### 3.3.1. Variations in Fluoride Release

**Figure 5:**
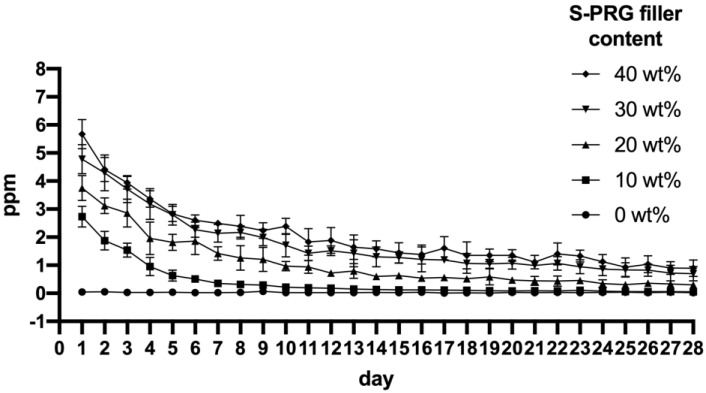
Fluoride ion release from APR disks containing 0, 10, 20, 30, and 40 wt% S-PRG filler, incubated in lactic acid solution for 28 d.

Figure 5 shows the fluoride release from APR disks containing the S-PRG filler. After 24 h, APR with 40 wt% S-PRG filler exhibited the highest amount of fluoride release (5.67 ppm), followed by 4.78 ppm for APR with 30 wt% S-PRG filler, 3.75 ppm for APR with 20 wt% S-PRG filler, and 2.73 ppm for APR with 10 wt% S-PRG filler. However, the fluoride release decreased rapidly on day 6, followed by a gradual decrease until day 14, and then remained constant during the 28 d period. The concentrations of the fluoride ions released from all disks exhibited similar trends at all measurement times, following the order of the S-PRG filler content. No fluoride release was observed in the APR without the S-PRG filler. Table 2 shows the statistical analysis of the fluoride ion release on days 1, 7, 14, 21, and 28.

#### 3.3.2. Al, B, Na, Si, and Sr Ion Release Profiles

**Figure 6:**
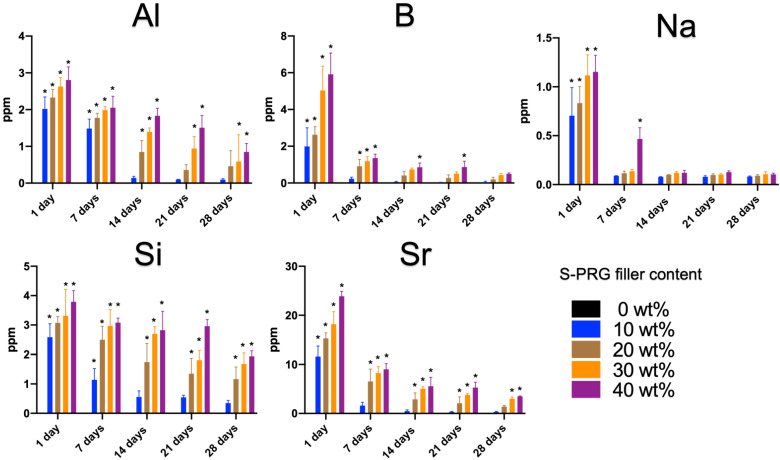
Ion release from APR containing the S-PRG filler after 1, 7, 14, 21, and 28 d. After 1 d, the release of all ions was significantly higher in APRs containing the S-PRG filler compared to the control (*p* < 0.05). The release of ions decreased over time on days 7, 14, 21, and 28. No ion was released in the APR with 0 wt% S-PRG filler; therefore, the y-axis coincided with 0 ppm. * *p* = 0.05.

Figure 6 shows the Al, B, Na, Si, and Sr ion release profiles obtained from the stock solution after 1, 7, 14, 21, and 28 d of incubation. The release of Al, B, Na, Si, and Sr ions from all the tested materials containing the S-PRG filler followed the order Sr > B > Si > Al > Na. All ions decreased as the exposure time increased. The amount of each released ion increased as the S-PRG filler content increased from 10 to 40 wt% after 24 h. Furthermore, the concentrations of the released ions decreased over time, as measured on days 14, 21, and 28.

### 3.4. Enamel Demineralization

**Figure 7:**
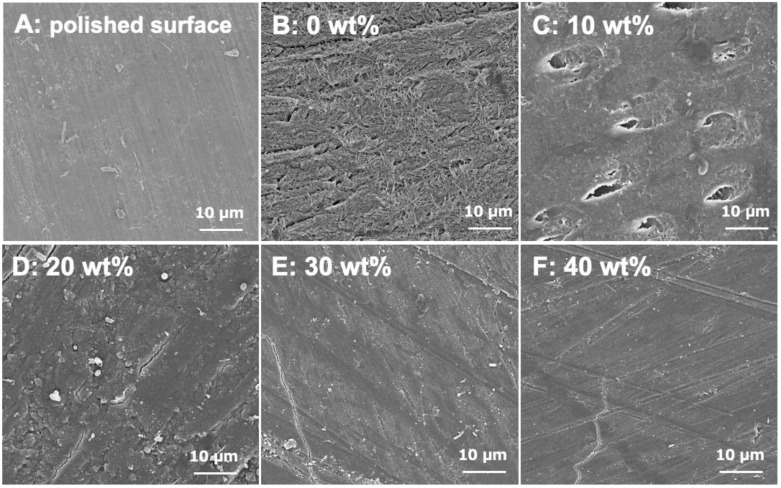
Representative SEM images of the enamel surface. (**A**) bovine enamel polished surface before immersion in lactic acid solution. (**B**–**F**) enamel surface attached with disks containing 0, 10, 20, 30, and 40 wt% S-PRG filler, respectively, immersed in lactic acid solution for 7 d (×3000).

Figure 7 shows the SEM images of the enamel block surface appearance of APRs containing 0, 10, 20, 30, and 40 wt% S-PRG fillers after 7 d of incubation. The pre-incubation enamel block surface exhibited a polished and smooth appearance with visible polishing lines and no signs of demineralization (Figure 7A). In contrast, in enamel blocks cultured with APR that did not contain the S-PRG fi2ller, the enamel surface was intensively demineralized with mineral loss (Figure 7B). The enamel block incubated with APR with 10 wt% S-PRG filler showed slightly demineralized enamel surfaces, representing an uncertain prism-like outline on the block surface due to demineralization (Figure 7C). Conversely, the enamel block surfaces in the culture of APRs with 20, 30, and 40 wt% S-PRG fillers showed no demineralized morphological surface appearance (Figure 7D–F), and their surfaces were similar to those of the polished control surfaces (Figure 7A).

## 4. Discussion

Denture cleaning methods using traditional chemical denture cleaners and mechanical methods are satisfactory and suitable for daily use at home; however, such methods remain inadequate for elderly and physically disabled patients. Denture-based resins incorporating antimicrobial and antifungal agents may be effective in eliminating biofilm formation and microbial adhesion [9]. In this study, we investigated a prosthetic system incorporating an S-PRG filler into an APR to develop a denture that exhibits biological activity and automatically maintains a healthy oral cavity. The characteristics of the S-PRG fillers include an acid-neutralizing effect, ion release, inhibition of enamel demineralization, and inhibition of *S. mutans* and fungal growth, which reduce the occurrence of early carious lesions and mucosal diseases [11,12]. Our results showed that APRs with S-PRG fillers neutralized low-pH lactic acid solutions (pH 4.0) to a neutral range (*p* < 0.05) and inhibited bovine enamel demineralization through the release of S-PRG filler ions at a content of 10 to 40 wt% S-PRG filler (*p* < 0.05). Therefore, the null hypothesis that this new APR formed with S-PRG filler would not exhibit sufficient bioactivity in terms of clinical performance was rejected. The results of this study suggest that the addition of S-PRG fillers into APR is effective for improving the biological activity with regard to ion release, pH neutralization, and inhibition of enamel demineralization. However, the incorporation of fillers into resins alters their physical and mechanical properties. To address this, we conducted the following experiments [20]: The flexural strength of APR containing the S-PRG filler (average particle size: 3 μm) decreased as the S-PRG filler content increased from 10 to 40 wt%, with the flexural strength of the APR containing less than 20 wt% S-PRG filler complying with the ISO requirements of exceeding 60 MPa [21]. Conversely, the flexural modulus of APR containing S-PRG fillers at all contents exceeded the minimum flexural modulus of 2.0 GPa specified by the ISO standard [21] for denture base resins. Kamijo et al. [22] investigated the mechanical properties of thermally polymerized polymethyl methacrylate (PMMA) with 5, 10, 20, 30, and 40 wt% S-PRG filler (average particle size: 4.1 μm). Our experimental flexural strength and flexural modulus results [20] are in agreement with Kamijo et al. [22], even though an auto-polymerizing resin (APR) was used in this study.

In our demineralization experimental model, the pH of the lactate solution was set to 4.0 and the experimental samples were incubated in the solution for 28 d, with the solution being renewed every 24 h. The pH of the lactic acid solution (pH 4.0) quickly increased to a neutral range when the resin disk containing the S-PRG filler came in contact with lactic acid solution, suggesting that the S-PRG filler has potential anticariogenic effects. It is very difficult to explain which ion is selectively or strongly effective for neutralization, or whether complicated interactions between ions are effective in acid buffering. However, our results demonstrated that the amount of each ion released from S-PRG showed a decreasing trend from the start of measurements until the 28th day (Figure 6), which is consistent with the tendency of the acid buffering capacity to gradually decrease with each daily measurement, resulting in a decrease in the pH value (Figure 4). By changing the solution to pH 4.0 every 24 h, the ions released from the S-PRG filler were used in the acid neutralization reaction; therefore, the ion storage capacity and acid buffering capacity gradually decreased over time. Additionally, resins containing 30 and 40 wt% S-PRG fillers released more ions than those containing 10 and 20 wt% S-PRG fillers, and their neutralizing effects continued for 28 d. Ion meter and ICP-OES measurements revealed that the Sr release was the highest, followed by F, B, Si, Al, and Na, showing that each ion concentration peaks at 24 h for each group and decreases owing to the release of ions over time. APR disks incorporating the S-PRG filler released the highest amount of fluoride on the first day of incubation, depending on the S-PRG filler content, and decreased daily following a similar trend, thus being released on day 28. Fluoride release and recharge treatments are the most widely used caries inhibition and remineralization methods for the initial stages of early enamel caries. The presence of free fluoride ions allows calcium and phosphate ions to be incorporated into the enamel crystal lattice, resulting in a fluorapatite mineral that is less soluble and more resistant to acid attacks and caries initiation [23]. A previous study reported that all the constituent ions Na, B, F, Sr, Si, and Al of the S-PRG filler were recharged when treated with a 9000 ppm NaF solution. This was demonstrated by the study investigating fluoride release and recharging capabilities of a fissure sealant incorporating the S-PRG filler [24]. Ion release from the S-PRG filler may suppress osteoclast formation and alveolar bone resorption caused by root caries [25]. The strontium released by the S-PRG filler may potentially integrate into the calcium positions within the enamel and dentin’s hydroxyapatite structure, potentially enhancing the strength of the tooth substrate. [26]. The S-PRG filler constantly buffers the acidic conditions owing to its ability to continuously release and recharge the ions constituting its structure. In addition, the S-PRG filler’s released ions are anticipated to be crucial in facilitating ionic interactions that promote tooth tissue remineralization in an acidic oral environment. Specifically, strontium is expected to work in conjunction with fluoride to remineralize or strengthen carious regions, such as enamel lesions that have undergone demineralization in the presence of fluoride. [27,28]. The addition of ions from the S-PRG filler to saliva has the potential to enhance the ionic saturation of saliva, facilitate ion penetration, and promote the incorporation of calcium and phosphate ions from saliva into the tooth surface [19]. Such reports suggest the possibility of tooth matrix remodeling.

Caries involves bacterial species such as *Streptococcus mutans*, *Lactobacillus*, *Actinomyces*, and others [29]. In studies on the antibacterial effects of fluoride and strontium on oral bacterial species, strontium exerts a synergistic antibacterial effect when present continuously in combination with fluoride [30]. The S-PRG filler inhibits glucose metabolism and the growth of *S. mutans*, and limits acid production owing to the antibacterial properties of the B and F ions [17,18]. Inflammation and erythema of the oral mucosa beneath dental prostheses is a condition termed denture stomatitis, which frequently manifests in individuals who utilize removable dental appliances. Given that *Candida albicans* is an opportunistic pathogen capable of colonizing both denture surfaces and oral mucosa [3], the finding that the S-PRG filler eluates inhibit the growth and metabolic formation of *Candida albicans* is noteworthy [31,32]. Furthermore, the ion release and recharge effects ensured that the antibacterial effects persisted for a long time. Therefore, dentures containing the S-PRG filler reduced *Candida albicans* adhesion and denture-induced stomatitis in the mucous membranes of denture wearers.

Embryologically, enamel development and mineralization are intricate processes tightly regulated by ameloblasts of the enamel organ. However, mature enamel is cell-free and, therefore, does not reconstruct itself using ameloblasts [7]. Notably, remineralization or apatite-like precipitation does not fully recover the original enamel structure. Nanosized S-PRG fillers can potentially improve periodontitis and bone healing in animal models. Moreover, nanosized S-PRG fillers showed inhibitory effects against biofilm-forming oral bacteria in in vitro studies [33]. Therefore, considering the results of several previous studies, it is possible to develop a long-lasting bioactive prosthesis using the S-PRG filler. To reduce the development of adherent biofilms, new bioactive denture materials with sufficient mechanical properties, biocompatibility, and aesthetics are being studied. Maintaining control over the oral microbiota is believed to prevent not only oral diseases, but also systemic diseases caused by the oral microbiota. Importantly, the addition of S-PRG fillers to dentures for oral applications affords dentures with long-lasting antibacterial effects.

A limitation of this study is the use of an in vitro design, affording different responses from those in actual in vivo conditions. However, the experiments were conducted in a lactic acid solution at pH 4.0, which is a harsher environment than the actual in vivo conditions. Further studies are needed to elucidate the effects of S-PRG fillers on tooth surfaces.

## 5. Conclusions

The developed auto-polymerizing resin containing the S-PRG filler is able to release ions and maintain a low-pH solution in the neutral range, thus inhibiting enamel demineralization. The effective use of the S-PRG filler in prosthetics as a bioactive material could prevent enamel demineralization.

## Figures and Tables

**Table 1:** pH values of the five S-PRG filler-containing auto-polymerizing resin disks after immersion for 1, 7, 14, 21, and 28 d in lactic acid solution.

S-PRG Filler Content (wt%)	Immersion Period (Days)				
	1	7	14	21	28
0 (Control)	4.01 ± 0.00	4.00 ± 0.00	4.01 ± 0.00	4 ± 0.00	4 ± 0.00
10	5.12 ± 0.15 *	4.09 ± 0.05	3.98 ± 0.01	3.98 ± 0.03	3.98 ± 0.06
20	5.56 ± 0.12 *	4.59 ± 0.24 *	4.15 ± 0.05	4.12 ± 0.13	4.07 ± 0.09
30	5.91 ± 0.11 *	4.92 ± 0.16 *	4.51 ± 0.16 *	4.42 ± 0.09 *	4.29 ± 0.12 *
40	6.09 ± 0.15 *	5.23 ± 0.18 *	4.69 ± 0.08 *	4.48 ± 0.13 *	4.42 ± 0.11 *

Two-way analysis of variance and Dunnett’s multiple comparison test (control = 0 wt% S-PRG filler content, * *p* < 0.05).

**Table 2:** Fluoride ion release after immersion in lactic acid solution for 1, 7, 14, 21, and 28 d.

S-PRG Filler Content (wt%)	Immersion Period (Days)				
	1	7	14	21	28
0 (Control)	0.0 ± 0.00	0.0 ± 0.00	0.0 ± 0.00	0.0 ± 0.00	0.0 ± 0.00
10	2.73 ± 0.32 *	0.36 ± 0.08	0.14 ± 0.04	0.09 ± 0.03	0.05 ± 0.01
20	3.75 ± 0.38 *	1.42 ± 0.21 *	0.60 ± 0.10	0.44 ± 0.14	0.30 ± 0.10
30	4.78 ± 0.45 *	2.14 ± 0.27 *	1.30 ± 0.31 *	0.99 ± 0.08 *	0.70 ± 0.22 *
40	5.67 ± 0.45 *	2.49 ± 0.18 *	1.59 ± 0.25 *	1.11 ± 0.21 *	0.90 ± 0.25 *

After 1 d, fluoride release was observed in all the resins containing the S-PRG fillers (*p* < 0.05). The released amount decreased over time, and after 28 d, the amount of release was significantly higher in the APR with 30 and 40 wt% S-PRG fillers than in the control group (*p* < 0.05). Two-way analysis of variance and Dunnett’s multiple comparison test (control = 0 wt% S-PRG filler content, * *p* < 0.05).

## Data Availability

The original contributions presented in the study are included in the article, further inquiries can be directed to the corresponding author.
